# Supplementary material for: Cryo-EM structure-based selection of computed ligand poses enables design of MTA-synergic PRMT5 inhibitors of better potency
Source: Commun Biol. 2022 Oct 3;5:1054. doi: 10.1038/s42003-022-03991-9 (PMC9530242; doi:10.1038/s42003-022-03991-9)
Supplement: Supplementary file 2 — Supplementary Information [file 42003_2022_3991_MOESM2_ESM.pdf]

Supplementary Information

Cryo-EM structure-based selection of computed ligand poses enables design of MTA-synergic PRMT5 inhibitors of better potency

Wei Zhou[1,2,&‡], Gaya P. Yadav[3,4,&‡], Xiaozhi Yang[1,2], Feng Qin[5], Chenglong Li[1,2,6,*], and Qiu-Xing Jiang[1,3,4,5,*#]

1. Department of Medicinal Chemistry, College of Pharmacy, University of Florida, Gainesville, FL 32610, United States.

2. Department of Biochemistry and Molecular Biology, College of Medicine, University of Florida, Gainesville, FL 32610, United States.

3. Department of Microbiology and Cell Science, University of Florida, 1355 Museum Drive, Gainesville, FL 32611, United States

4. Laboratory of Molecular Physiology and Biophysics, Hauptmann-Woodward Medical Research Institute, Buffalo, NY 14203, United States.

5. Department of Physiology and Biophysics, University at Buffalo, Buffalo 14214, United States.

6. Center for Natural Products, Drug Discovery and Development (CNPD3), University of Florida, Gainesville, FL 32610, United States.

& Current addresses: (G.P.Y.): Department of Biochemistry and Biophysics, Texas A &M University, College Station, TX 77843, United States.

List of supplementary items:

Supplementary Figures 1-9

Supplementary Tables 1-7

Supplementary References

Supplementary Figures and legends:

Supplementary Figure 1. PRMT5:MEP50 complex purification and characterization.

a). Size exclusion chromatography of the PRMT5:MEP50 complex after anti-FLAG affinity column. Fractions at the indicated area were collected and concentrated. **b).** An SDS-PAGE gel is shown for the collected fractions of PRMT5:MEP50 with a purity greater than 95%. **c).** Sedimentation velocity analytical ultracentrifugation (SV-AUC). The PRMT5:MEP50 complex has an estimated mass of 445 kDa.

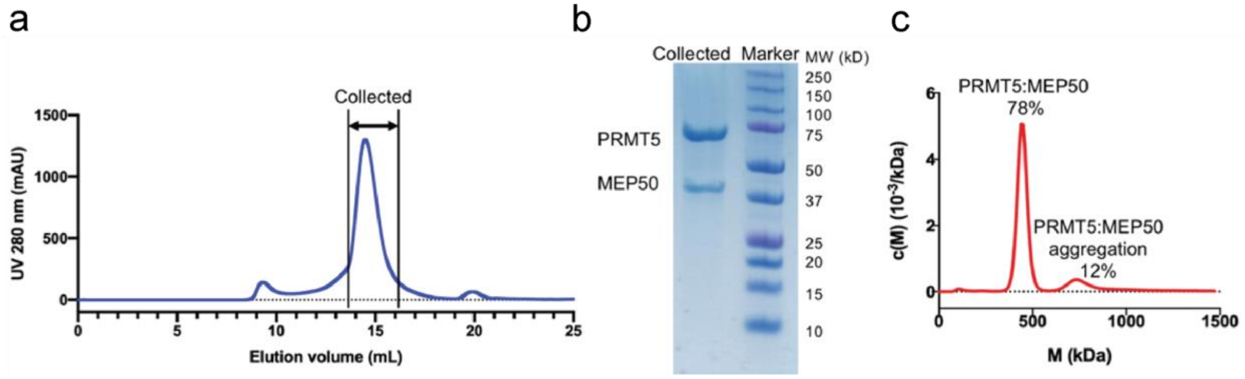

Supplementary Figure 2. Processing cryo-EM data of the PRMT5: MEP50/ 11-2F/ MTA complex. The final map has an overall nominal resolution of 3.14 Å (FSC=0.143). The presentations were prepared in Chimera [1] and PyMOL[2].

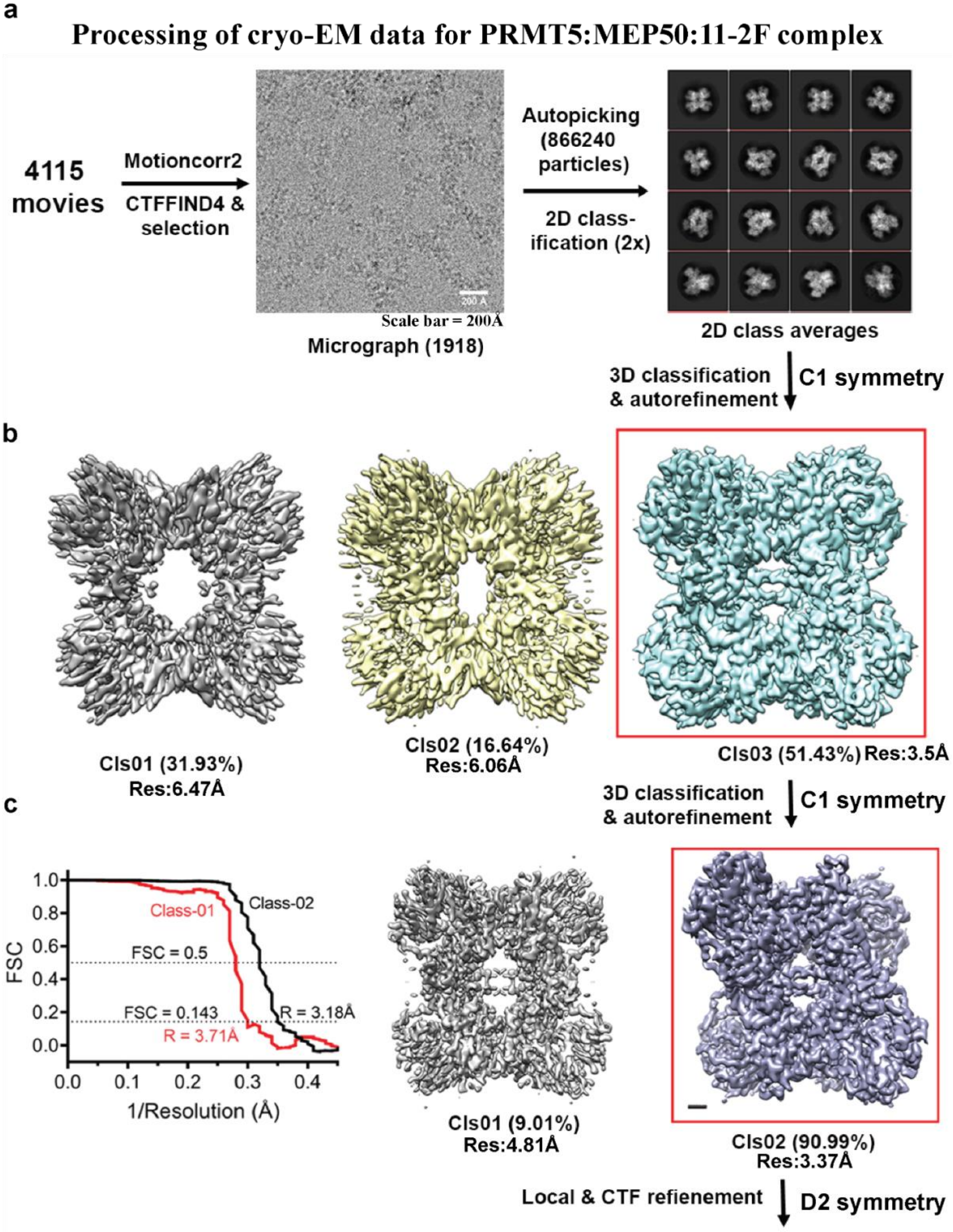

d

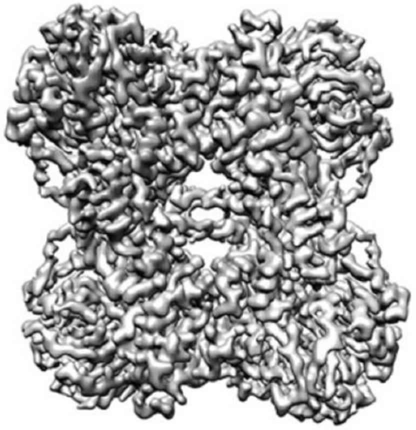

Final map (207392 particles)
(Res = 3.14Å, D2 symmetry)

e

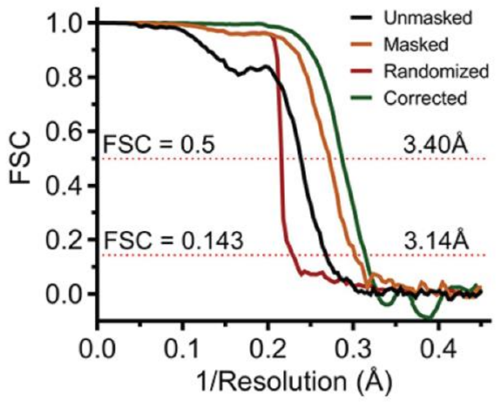

FSC curve

f

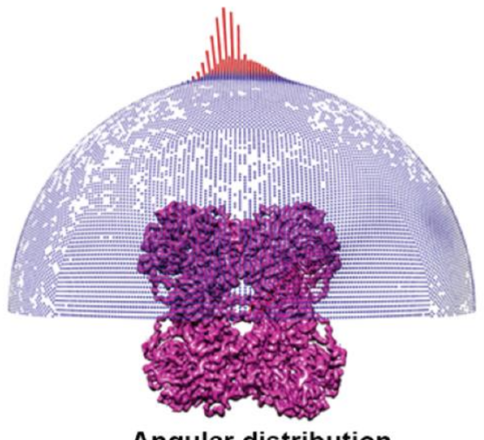

Angular distribution

Supplementary Figure 3. Top poses of different ligands from computational analysis

a). Top 8 poses of MTA. In each of the 2,000 runs, 25 million seed positions were used as starting points for movements and energy minimization. The top poses from 2,000 runs were clustered based on their RMSD. The best ones of the resultant 8 clusters are showed.

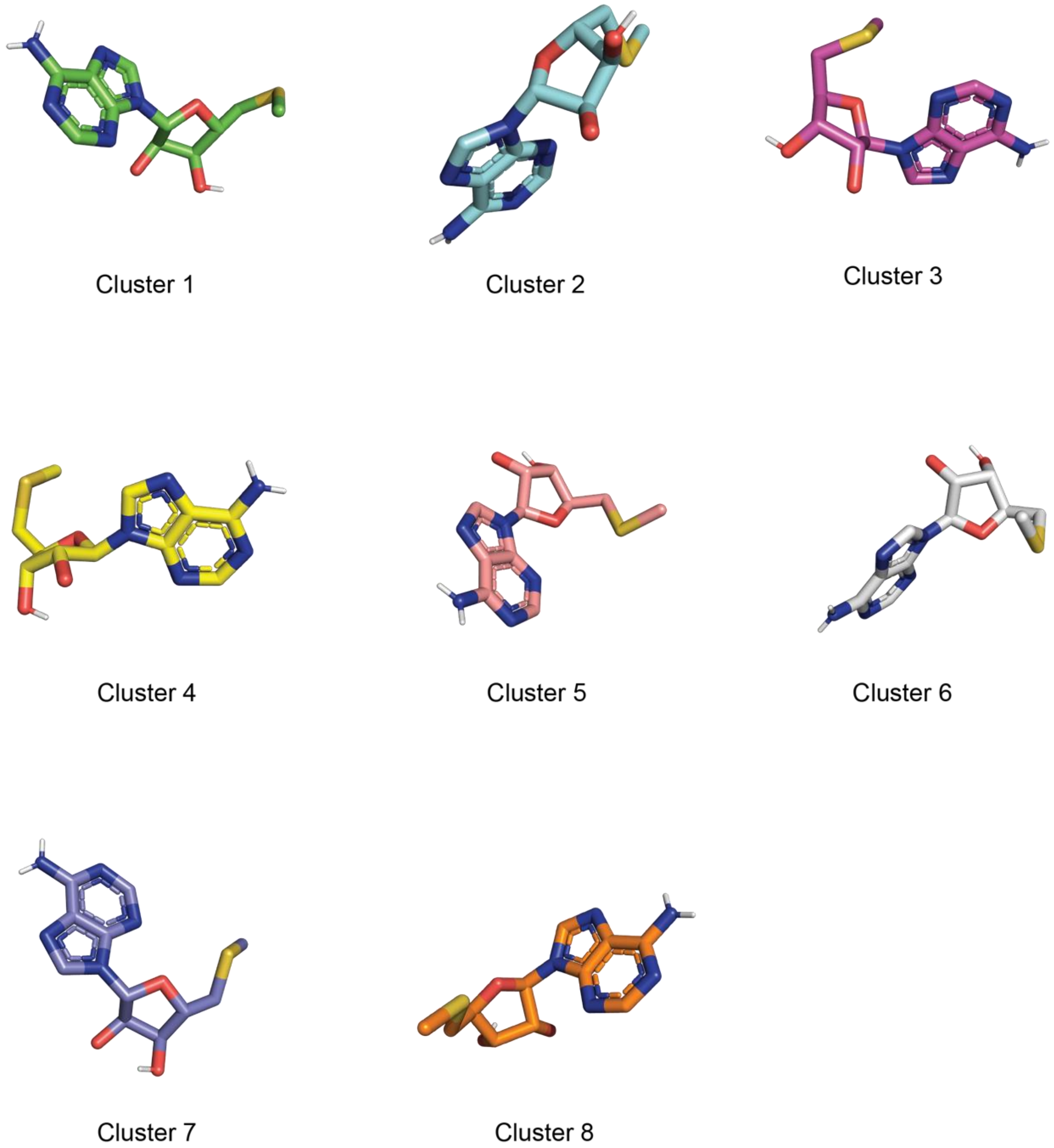

b). Top 10 poses of SAH came from analysis as explained in a.

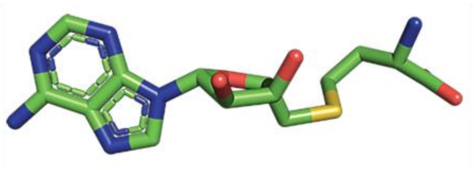

Cluster 1

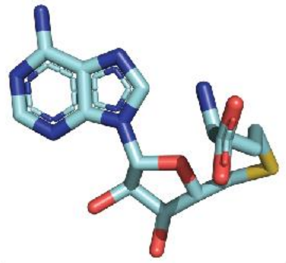

Cluster 2

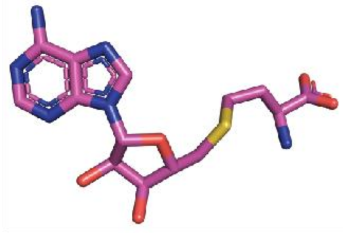

Cluster 3

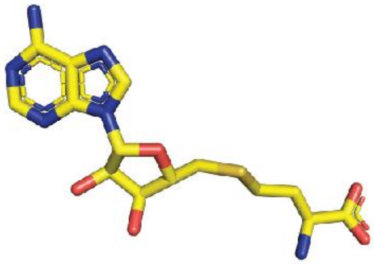

Cluster 4

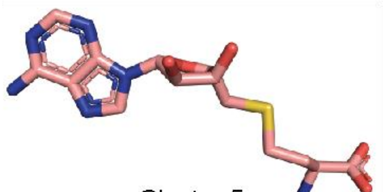

Cluster 5

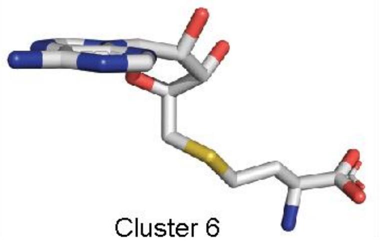

Cluster 6

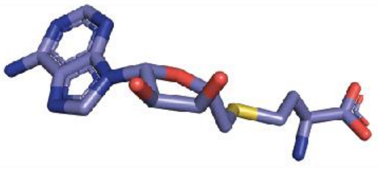

Cluster 7

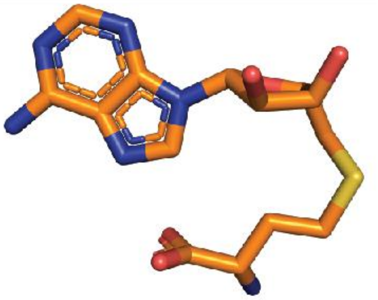

Cluster 8

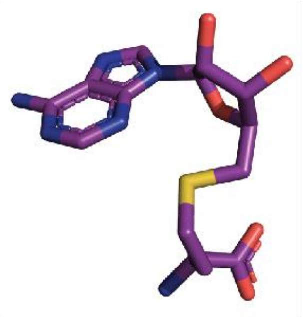

Cluster 9

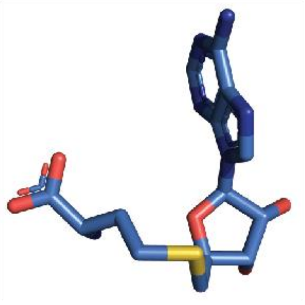

Cluster 10

c). Top 10 poses of SAM.

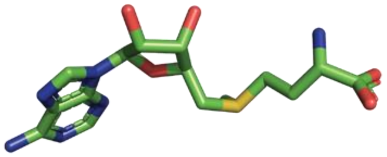

Cluster 1

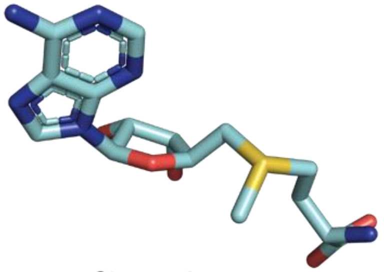

Cluster 2

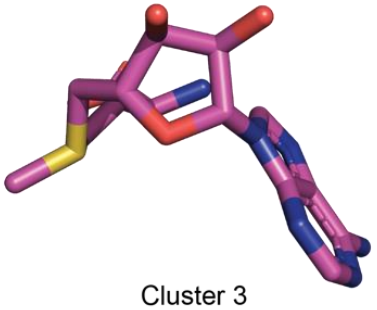

Cluster 3

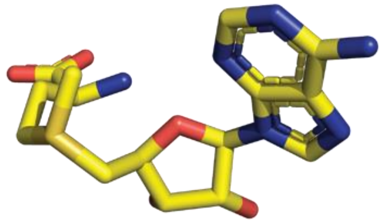

Cluster 4

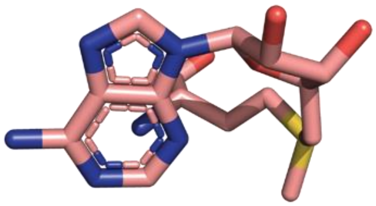

Cluster 5

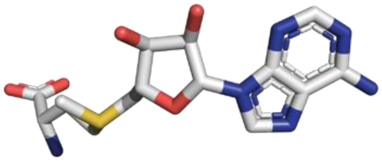

Cluster 6

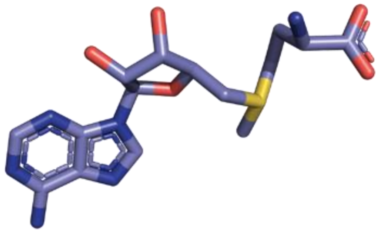

Cluster 7

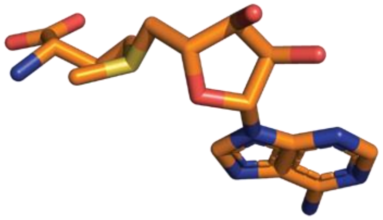

Cluster 8

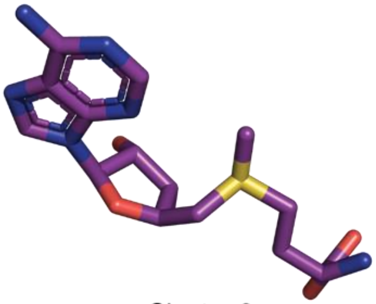

Cluster 9

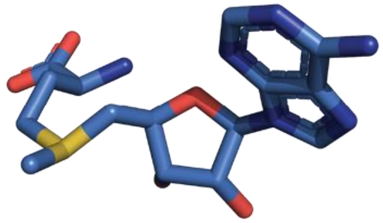

Cluster 10

d). Top 10 poses of 11-2F.

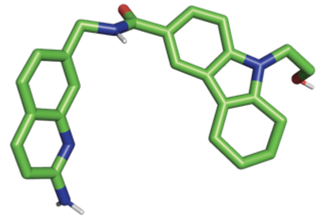

Cluster 1

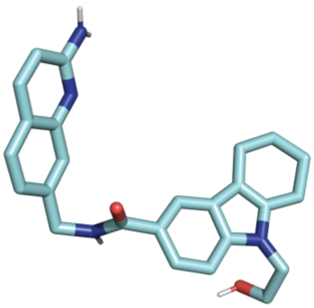

Cluster 2

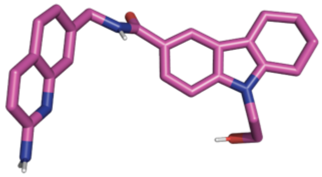

Cluster 3

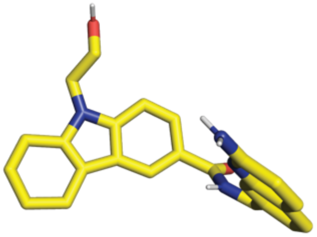

Cluster 4

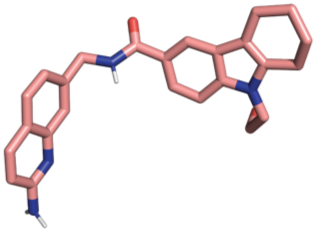

Cluster 5

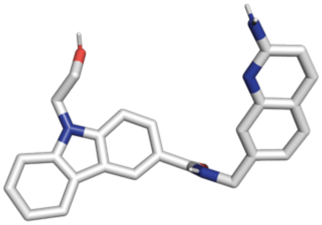

Cluster 6

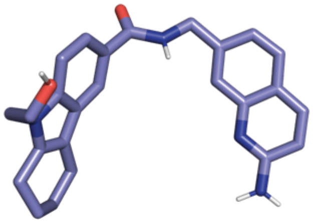

Cluster 7

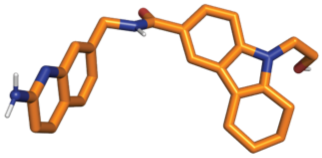

Cluster 8

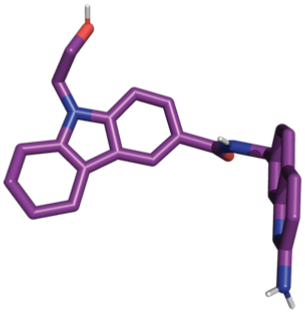

Cluster 9

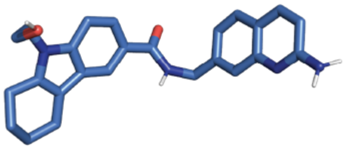

Cluster 10

e). Two opposite orientations of 11-2F differ in docking energy. The refined pose in yellow and the other pose representing cluster 4 (white) are fitted into the cryo-EM density map of the ligand (semitransparent grey). The two had their head and tail parts in opposite arrangements and were not distinguished reliably by fitting ligand atoms into the cryo-EM density at the current 3.1 Å resolution.

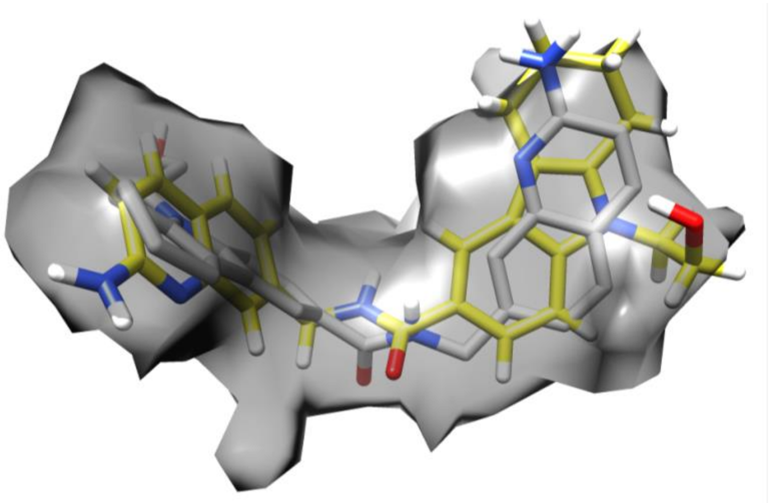

f). Two opposite orientations of 11-2F inside the binding pocket. The residues in the binding pocket (blue mesh) did not change significantly to accommodate the two poses. The refinement statistics in PHENIX for PRMT5 and ligands with 11-2F in two opposite orientations in the binding pocket (cyan vs. pink) were the same as in Table 1.

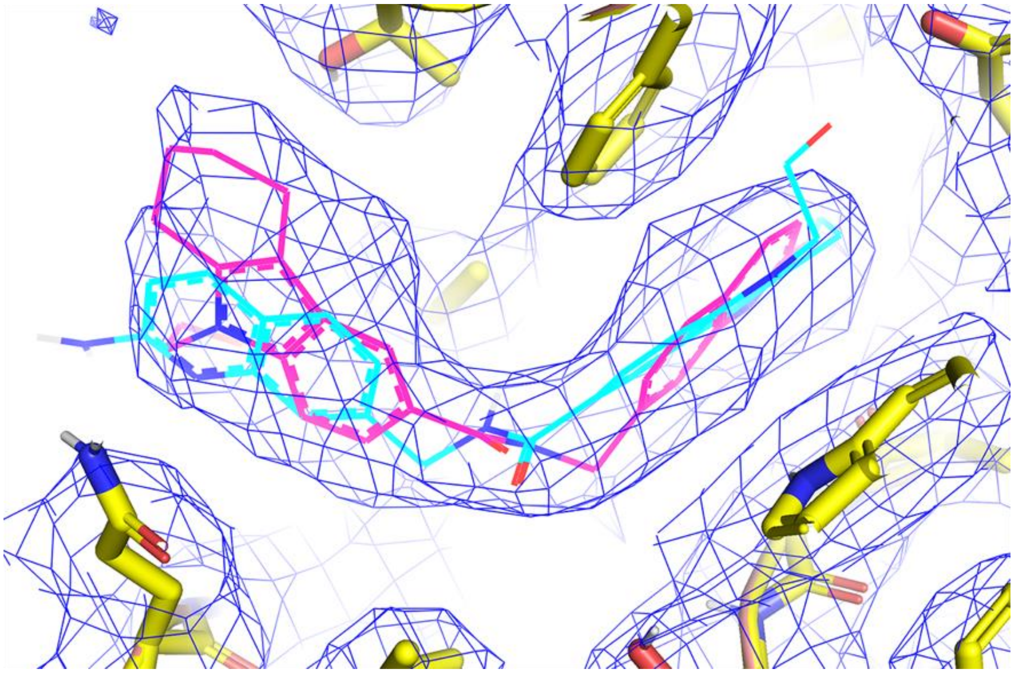

Supplementary Figure 4. Processing cryo-EM data of the *apo* PRMT5: MEP50 complex. The final map is of 3.2 Å (FSC = 0. 143). Presentations were prepared in Chimera [1] and PyMOL [2].

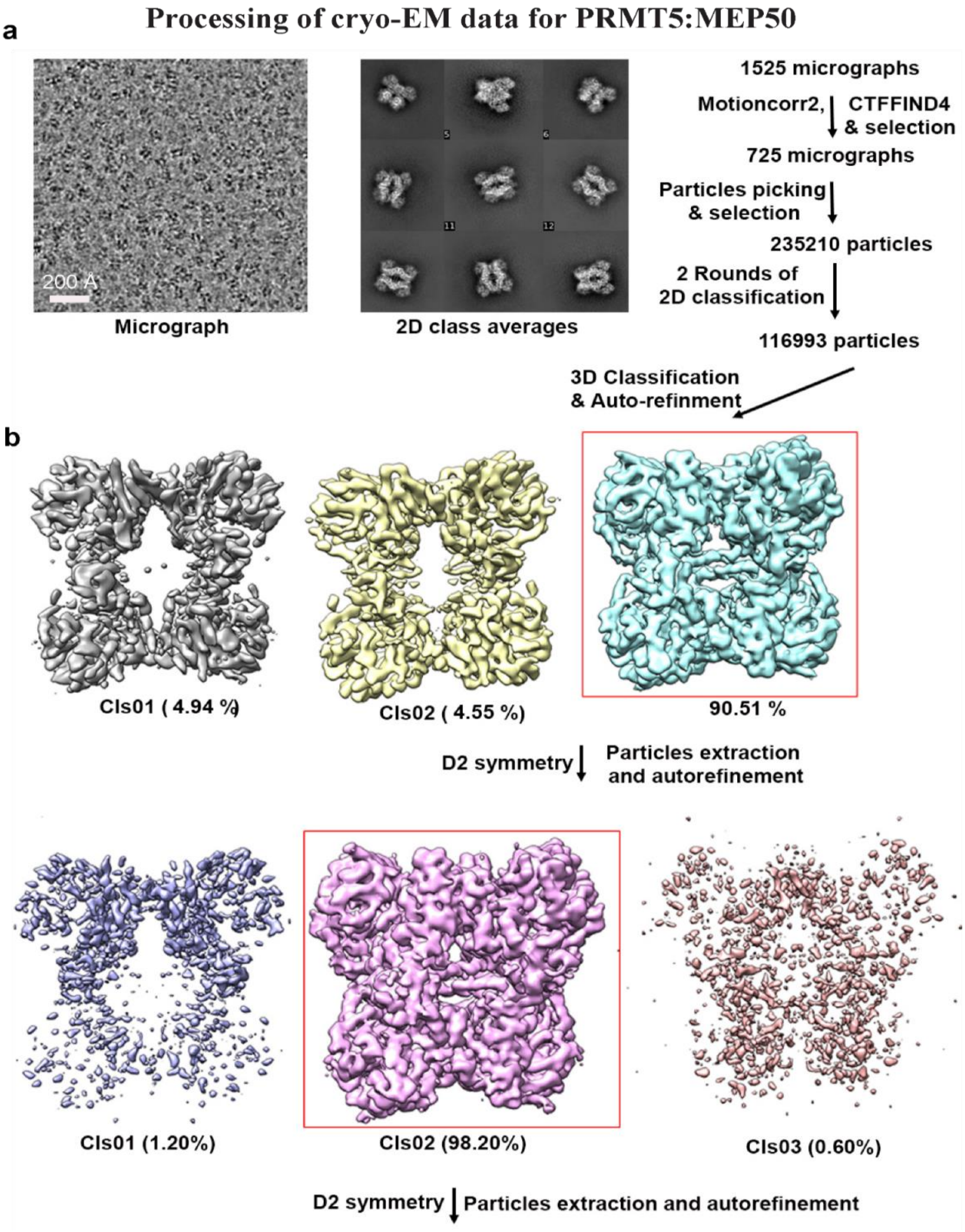

c

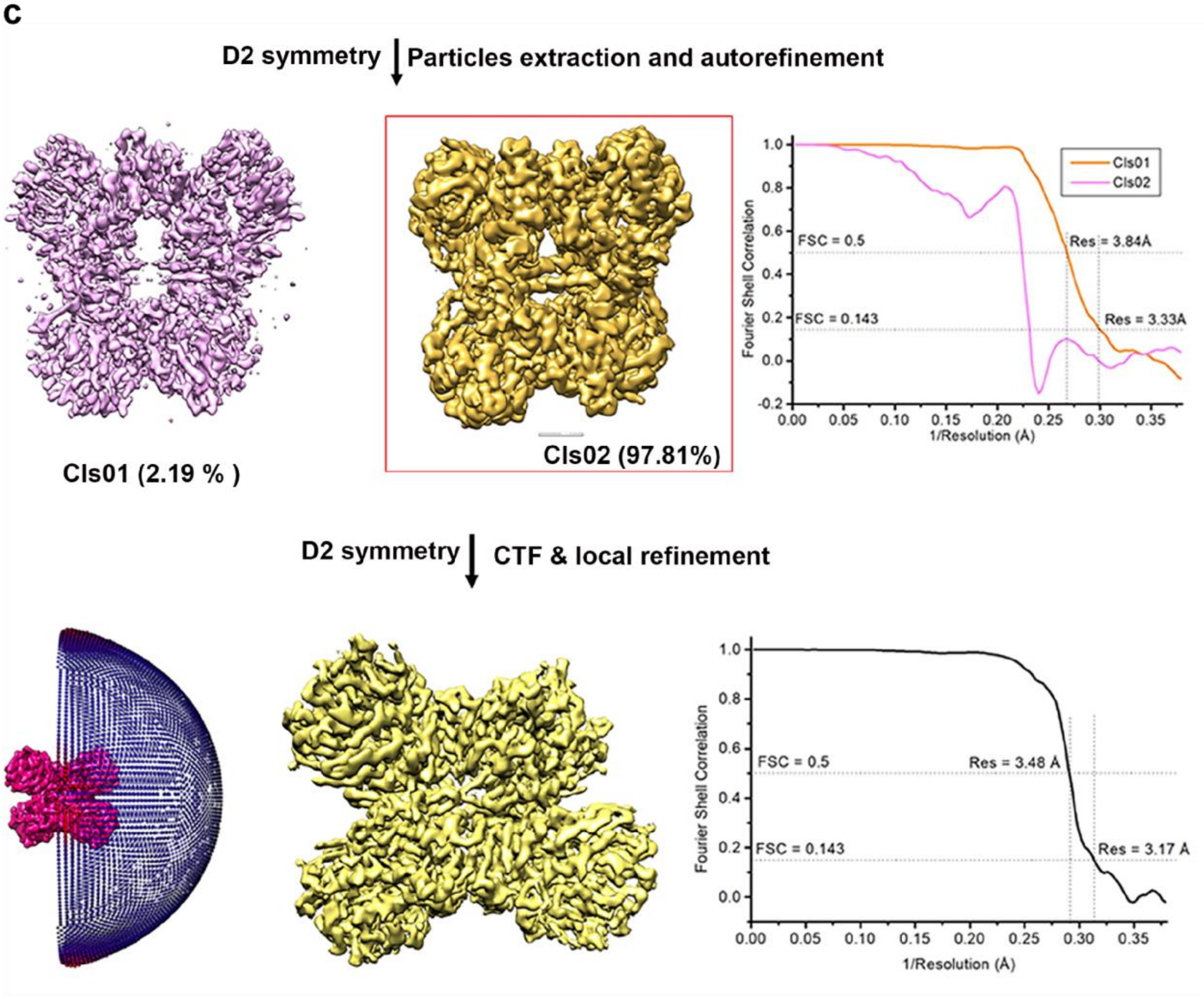
D2 symmetry
Particles extraction and autorefinement
Cls01 (2.19 %)
Cls02 (97.81%)
Cls01
Cls02
Fourier Shell Correlation
FSC = 0.5
FSC = 0.143
Res = 3.84Å
Res = 3.33Å
1/Resolution (Å)
D2 symmetry
CTF & local refinement
Fourier Shell Correlation
FSC = 0.5
FSC = 0.143
Res = 3.48 Å
Res = 3.17 Å
1/Resolution (Å)

Supplementary Figure 5. Sequence alignment of PRMT1 - 7 focusing on their catalytic domains. Key residues conserved among the catalytic domains are highlighted (*, :).

```
hPRMT7   LHDKDRNVKYYQGIRAAV----SRVKDRGQKALVLDIGTGTGLLSMM----AVTAG-ADF   89
hPRMT5   EKDPIKYSQYQQAIYKCLLDRVPEEEKDTNVQVLMVLGAGRGPLVNASLRAAKQADRRIK   387
hPRMT1   LKDEVRTLTYRNSMFHNR--------HLFKDKVVLDVGSGTGILQMF----AAKAG-ARK   113
hPRMT3   LKDKIRTESYRDFIYQNP--------HIFKDKVVLDVGCGTGILSMF----AAKAG-AKK   280
rPRMT4   MQDYVRTGTYQRAILQNH--------TDFKDKIVLDVGCGSGILSFF----AAQAG-ARK   210
hPRMT2   LADQPRTTKYHSVILQNK--------ESLTDKVILDVGCGTGIISLF----CAHYARPRA   163
hPRMT6   IADRVRTDAYRLGILRNW--------AALRGKTVLDVGAGTGILSIF----CAQAG-ARR   107
           *  :   *   :                   :: :* * * :       .

hPRMT7   CYAIEVFKPMADAAVKIVEKNGFSDKIKVINKHSTEVTVGPEGDMPCRANILVTELFDTE   149
hPRMT5   LYAVEK-NPNAVVTLENWQFEEWGSQVTVVSSDMREWVAP------EKADIIVSELLGSF   440
hPRMT1   VIGIEC-SSISDYAVKIVKANKLDHVVTIIKGKVEEVELPV-----EKVDIIISEWMGYC   167
hPRMT3   VLGVDQ-SEILYQAMDIIRLNKLEDTITLIKGKIEEVHLPV-----EKVDVIISEWMGYF   334
rPRMT4   IYAVEA-STMAQHAEVLVKSNNLTDRIVVIPGKVEEVSLP------EQVDIIISEPMGYM   263
hPRMT2   VYAVEA-SEMAQHTGQLVLQNGFADIITVYQQKVEDVVLP------EKVDVLVSEWMGTC   216
hPRMT6   VYAVEA-SAIWQQAREVVRFNGLEDRVHVLPGPVETVELP------EQVDAIVSEWMGYG   160
           .::  .     :      :     : :                  :.: :::* :.

hPRMT7   LIGEGALPSYEHAHRHLVEENCEAVPHRATVYAQLVESGRMWSW---NKLFPIHVQTSLG   206
hPRMT5   ADNELSPE-CLDGAQHFLKDDGVSIPGEYTSFLAPISSSKLYNEVRACREKDRDP--EAQ   497
hPRMT1   LFYESMLNTVLYARDKWLAPDGLIFPDRATLYVTAIEDRQYKDY---KIHWWENV---YG   221
hPRMT3   LLFESMLDSVLYAKNKYLAKGGSVYPDICTISLVAVSDVNKHAD---RIAFWDDV---YG   388
rPRMT4   LFNERMLESYLHAK-KYLKPSGNMFPTIGDVHLAPFTDEQLYMEQFTKANFWYQPS-FHG   321
hPRMT2   LLFEFMIESILYARDAWLKEDGVIWPTMAALHLVPCSADKDYRS---KVLFWDNA---YE   270
hPRMT6   LLHESMLSSVLHARTKWLKEGGLLLPASAELFIAPISDQ-MLEW---RLGFWSQVKQHYG   216
            *        .    : .    *                           .

hPRMT7   ------------EQVIVPPVDVESCP----GAPSVCDIQLNQVSPADFTVLSDVLPMFSI   250
hPRMT5   FEMPYV---------------VRLHNFHQLSAPQPCFTFSHPN--RDPMIDN----NRYC   536
hPRMT1   FDMSCIKDVAIKEPL------VDVVDPKQ-LVTNACLIKEVDI--YTVKVED---LTFTS   269
hPRMT3   FKMSCMKKAVIPEAV------VEVLDPKT-LISEPCGIKHIDC--HTTSISD---LEFSS   436
rPRMT4   VDLSALRGAAVDEYFRQPVVDTFDIRI---LMAKSV---KYTV--NFLEAKEGDLHRIEI   373
hPRMT2   FNLSALKSLAVKEFFSKPKY-NHILKPED-CLSEPCTILQLDM--RTVQIS--DLETLRG   324
hPRMT6   VDMSCLEGFATRCLMGHSEIVVQGLSGED-VLARPQRFAQLEL--SRAGLEQELEAGVGG   273

hPRMT7   DFSKQVSSSAACHSRRFEPLTSGRAQVVLSWWDIEMDPEGKIKCTMAPFWAHSDPE-EMQ   309
hPRMT5   TLE-------------FPVEVNTVLHGFAGYFETVLYQD---------ITLSIRPETHSP   574
hPRMT1   PFC-------------LQVKRNDYVHALVAYFNIEFT-R----CH-KRTGFSTSPE---S   307
hPRMT3   DFT-------------LKITRTSMCTAIAGYFDIYFEKN----CH-NRVVFSTGPQ---S   475
rPRMT4   PFK-------------FHMLHSGLVHGLAFWFDVAFIGS-----I-MTVWLSTAPT---E   411
hPRMT2   ELR-------------FDIRKAGTLHGFTAWFSVHFQSLQ--EGQ-PPQVLSTGPF---H   365
hPRMT6   RFR-------------CSCYGSAPMHGFAIWFQVTFPGG---ESE-KPLVLSTSPF---H   313
          :                         .  ::.  :                  *

hPRMT7   WRDHWMQCVYFLPQEEPVVQGSALYLVA-------HHDDYCVWYSLQRTSPEKNERVRQM   362
hPRMT5   GMFSWFPILFPIKQPITVREGQTICVRFWRCSNS-----KKVWYEWAVTAPVCSAIHNP-   628
hPRMT1   PYTHWKQTVFYMEDYLTVKTGEEIFGTIGMRPNAKNNRDLDFTIDLDFKGQLCELSCST-   366
hPRMT3   TKTHWKQTVFLLEKPFSVKAGEALKGKVTVHKNKKDPRSLTVTLTLNNSTQTYGLQ----   531
rPRMT4   PLTHWYQVRCLFQSPLFAKAGDTLSGTCLLIANKRQSYDISIVAQVDQT---GSKSSNLL   468
hPRMT2   PTTHWKQTLFMMDDPVPVHTGDVVTGSVVLQRNPVWRRHMSVALSWAVTSRQDPTSQKVG   425
hPRMT6   PATHWKQALLYLNEPVQVEQDTDVSGEITLLPSRDNPRRLRVLLRYKVGDQEEKTKDFAM   373
             *      : .   .  .  :                 .
```

Supplementary Figure 6. Top 10 poses of 11-9F from computational analysis.

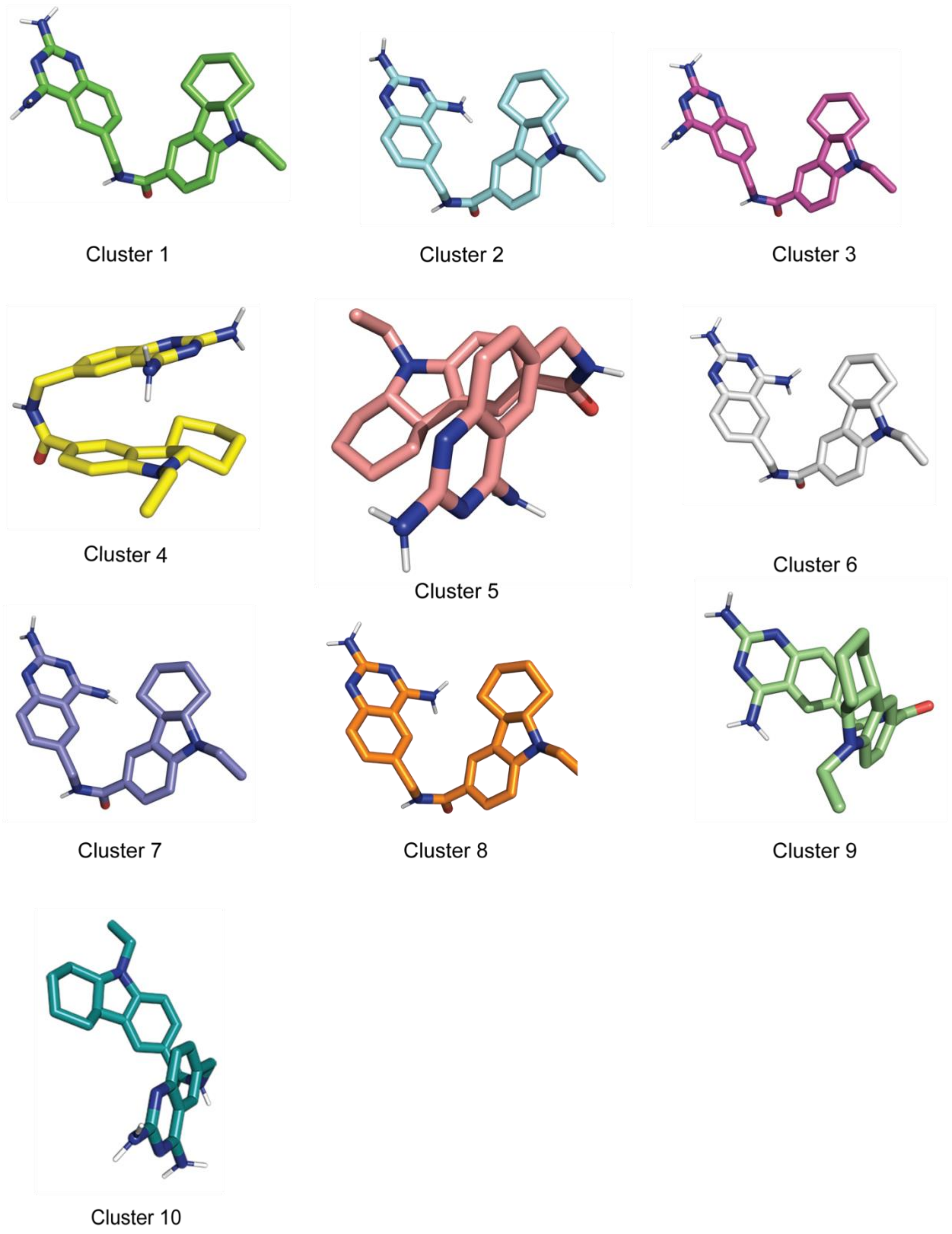

Supplementary Figure 7. Top 10 poses of HWIem2104 from computational analysis.

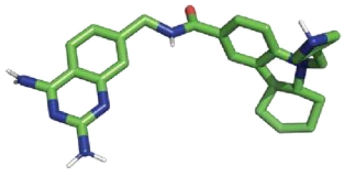

Cluster 1

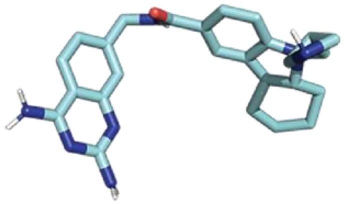

Cluster 2

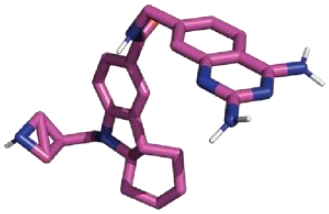

Cluster 3

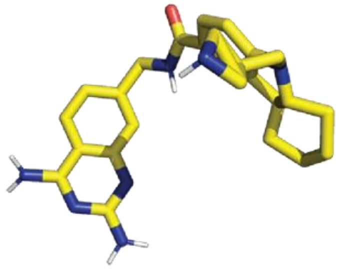

Cluster 4

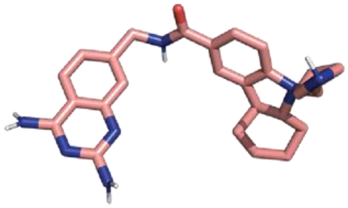

Cluster 5

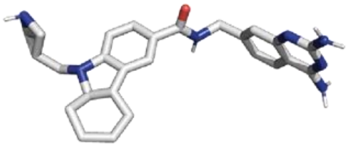

Cluster 6

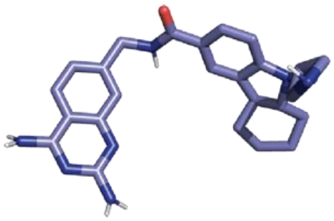

Cluster 7

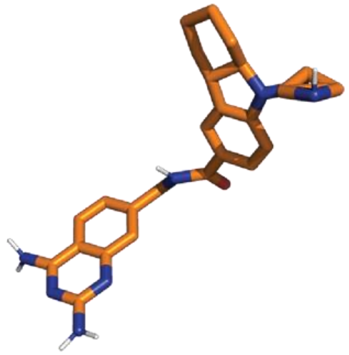

Cluster 8

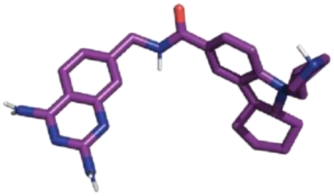

Cluster 9

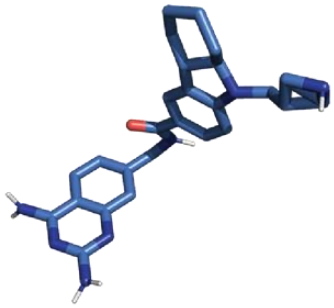

Cluster 10

Supplementary Figure 8. Top 10 poses of HWIem2109 from computational analysis.

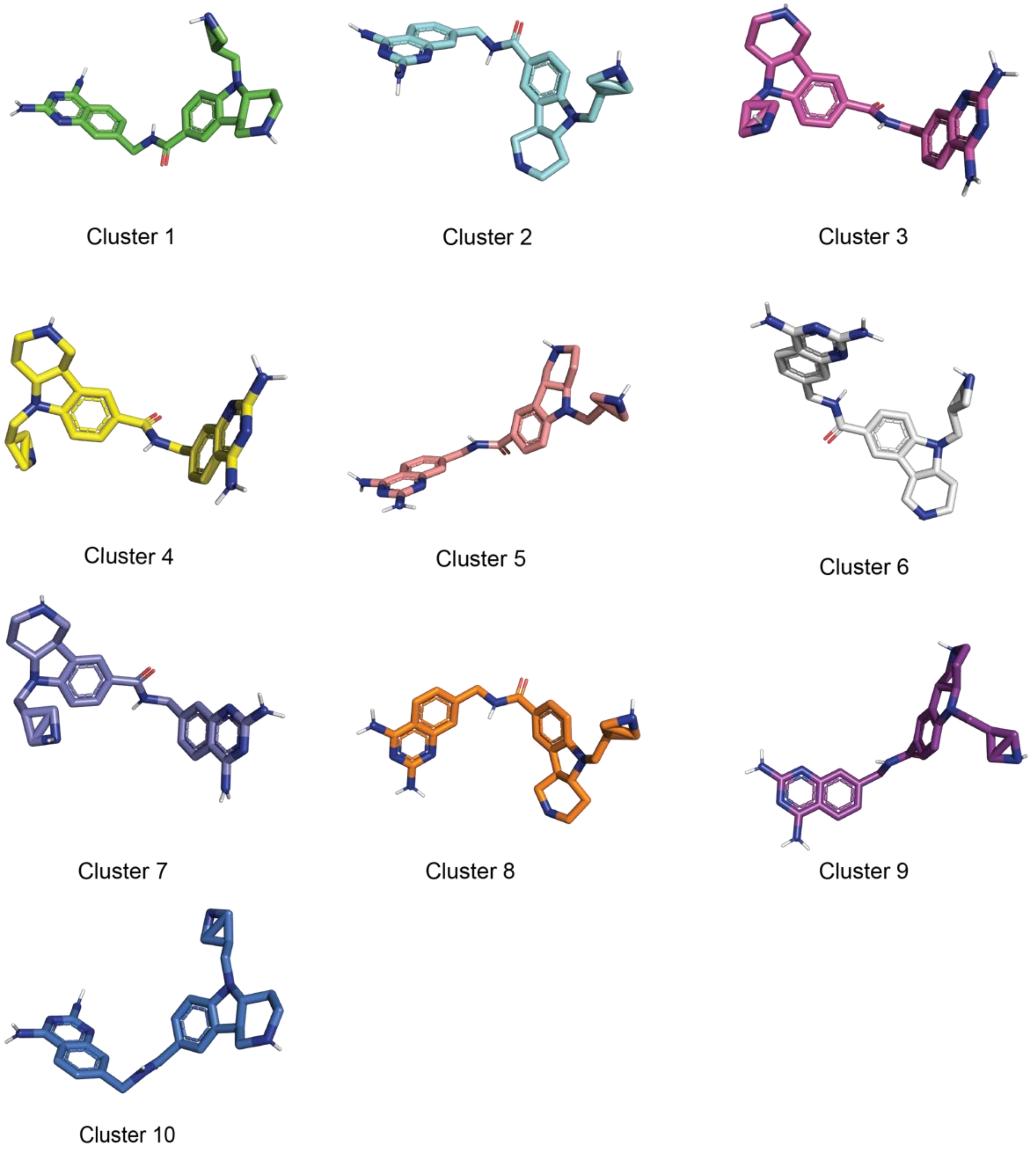

Supplementary Figure 9. Uncropped image from western blotting of SDMA

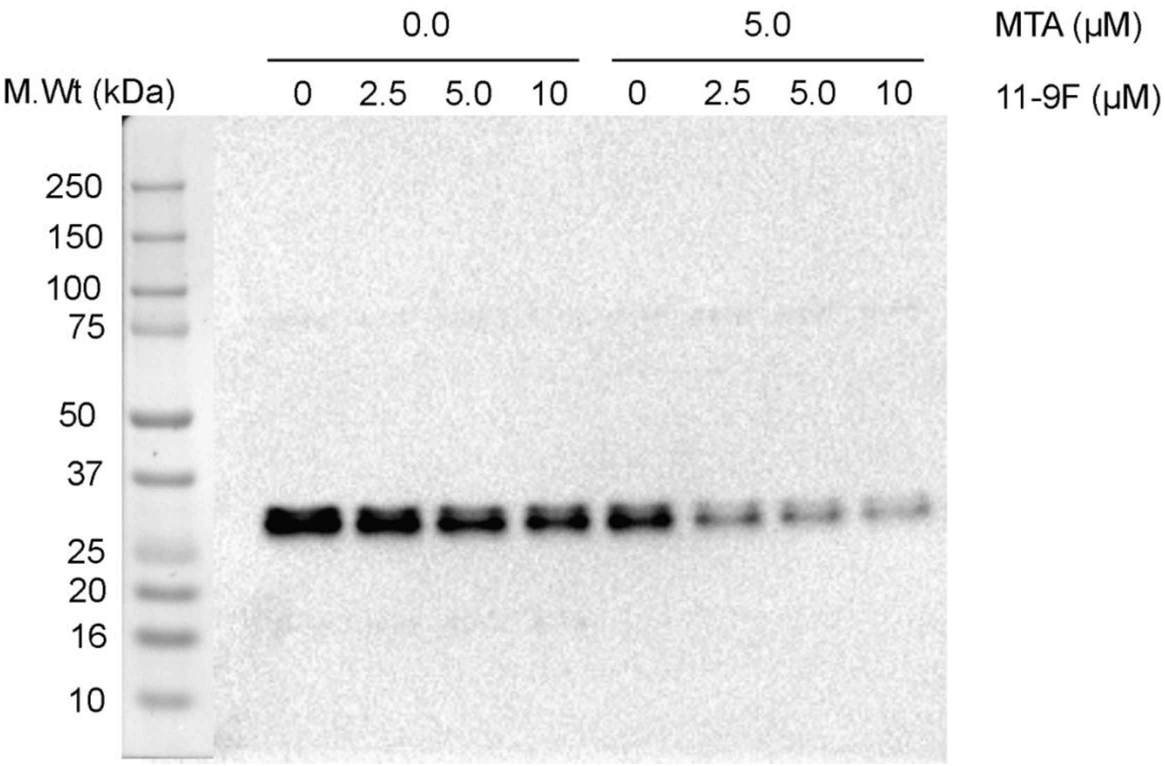

Supplementary Tables 1- 7:

Supplementary Table 1. Clusters of energy-minimized poses for MTA

Clus -ter Rank	Lowest Binding Energy	Run	Mean Binding Energy	Num in Clus
1	-6.63	1556	-6.03	1781
2	-5.95	241	-5.39	104
3	-5.62	1994	-5.54	48
4	-5.49	1083	-5.45	32
5	-5.21	1555	-5.17	4
6	-5.18	107	-5.18	1
7	-5.06	526	-4.94	24
8	-4.97	837	-4.97	6

Supplementary Table 2. Clusters of energy-minimized poses for SAH

Clus -ter Rank	Lowest Binding Energy	Run	Mean Binding Energy	Num in Clus
1	-8.80	1101	-8.13	346
2	-8.64	1726	-7.88	163
3	-8.46	9	-7.91	177
4	-8.30	694	-7.82	151
5	-8.28	496	-7.69	227
6	-7.82	353	-7.36	162
7	-7.78	1750	-7.53	24
8	-7.64	656	-7.33	15
9	-7.59	520	-6.97	241
10	-7.52	1639	-6.81	39

Supplementary Table 3. Clusters of energy-minimized poses for SAM

Cluster Rank	Lowest Binding Energy	Run	Mean Binding Energy	Num in Clus
1	-8.65	1187	-8.22	112
2	-8.55	1520	-8.26	128
3	-8.12	276	-8.15	197
4	-7.99	1993	-7.93	256
5	-7.80	1899	-7.73	373
6	-7.80	653	-7.72	3
7	-7.75	1018	-7.71	50
8	-7.38	129	-7.17	30
9	-7.27	1644	-7.53	22
10	-7.17	1824	-7.51	3

Supplementary Table 4. Clusters of energy-minimized poses for 11-2F

Cluster Rank	Lowest Binding Energy	Run	Mean Binding Energy	Num in Clus
1	-10.99	301	-10.18	49
2	-10.92	825	-10.43	70
3	-10.38	875	-9.72	10
4	-10.38	1353	-9.68	540
5	-10.36	234	-9.68	24
6	-10.21	512	-9.74	173
7	-10.13	304	-9.67	20
8	-10.10	1757	-9.28	11
9	-9.92	1551	-9.37	22
10	-9.89	217	-9.28	114

Supplementary Table 5: Clusters of energy-minimized poses for 11-9F

Cluster Rank	Lowest Binding Energy	Run	Mean Binding Energy	Num in Clus
1	-10.95	1852	-10.43	1120
2	-10.80	42	-10.47	93
3	-10.64	1780	-10.58	4
4	-10.59	1386	-10.24	3
5	-10.25	626	-9.84	20
6	-10.09	68	-9.86	10
7	-10.08	1179	-9.93	20
8	-10.07	110	-9.33	17
9	-10.03	18	-9.84	23
10	-10.02	1457	-9.88	14

Supplementary Table 6. Clusters of energy-minimized poses for HWIem2104

Cluster Rank	Lowest Binding Energy	Run	Mean Binding Energy	Num in Clus
1	-12.67	968	-11.98	1117
2	-12.64	1222	-11.54	160
3	-11.60	6	-10.62	284
4	-11.29	531	-10.17	4
5	-11.24	394	-10.75	14
6	-11.23	23	-10.57	5
7	-10.93	1715	-10.93	1
8	-10.82	446	-10.53	34
9	-10.63	1067	-10.31	15
10	-10.23	1373	-10.03	10

Supplementary Table 7. Clusters of energy-minimized poses for HWIem2109

Clus-ter Rank	Lowest Binding Energy	Run	Mean Binding Energy	Num in Clus
1	-12.26	725	-11.56	420
2	-11.69	879	-11.06	290
3	-10.98	1254	-10.48	911
4	-10.83	631	-10.14	44
5	-10.28	1034	-9.81	58
6	-10.27	1291	-9.72	21
7	-10.24	1561	-9.96	173
8	-10.22	169	-10.22	1
9	-10.21	939	-9.86	6
10	-10.12	754	-10.12	1

Supplementary References:

1 Pettersen, E. F. *et al.* UCSF Chimera--a visualization system for exploratory research and analysis. *J Comput Chem* **25**, 1605-1612, doi:10.1002/jcc.20084 (2004).

2 The PyMOL Molecular Graphics System. v. 2.0 (Schrodinger, LLC, 2020).
