# Supplementary material for: Cryo-EM structure-based selection of computed ligand poses enables design of MTA-synergic PRMT5 inhibitors of better potency
Source: Commun Biol. 2022 Oct 3;5:1054. doi: 10.1038/s42003-022-03991-9 (PMC9530242; doi:10.1038/s42003-022-03991-9)
Supplement: Supplementary file 4 — Author List Change [file 42003_2022_3991_MOESM4_ESM.pdf]

In accordance with Springer Nature Authorship Policy we agree to change the authors of the manuscript as indicated below.

NAME OF JOURNAL: Communications Biology

TITLE OF MANUSCRIPT: Cryo-EM structure-based selection of computed ligand poses ena

MANUSCRIPT NUMBER: COMMSBIO-21-3348D

CORRESPONDING AUTHORS NAME: Qiu-Xing Jiang and Chenglong Li

PREVIOUS AUTHOR NAMES:

Wei Zhou1,2,‡, Gaya P. Yadav3,4,&‡, Xiaozhi Yang1,2, Chenglong Li1,2,5,*, and Qiu-Xing Jiang1,3,4,6,*#

UPDATED AUTHOR NAMES:

Wei Zhou1,2,‡, Gaya P. Yadav3,4,&‡, Xiaozhi Yang1,2, Feng Qin5, Chenglong Li1,2,6,*, and Qiu-Xing Jiang1,3,4,5,*#

CHANGE TO AUTHOR LIST:

addition of Dr. Feng Qin

Print Name	Signature	Date
Wei Zhou	[illegible]	9/9/2022
Gaya P. Yadav	[illegible]	09/09/2022
Xiaozhi Yang	[illegible]	09/09/2022
Chenglong Li	Chenglong Li	9/9/2022
Qiu-Xing Jiang	[illegible]	9/2/2022
